# Supplementary material for: Regulation of Zbp1 by miR-99b-5p in microglia controls the development of schizophrenia-like symptoms in mice
Source: EMBO J. 2024 Mar 25;43(8):3. doi: 10.1038/s44318-024-00067-8 (PMC11021462; doi:10.1038/s44318-024-00067-8)
Supplement: Supplementary file 1 — Appendix [file 44318_2024_67_MOESM1_ESM.pdf]

Regulation of Zbp1 by miR-99b-5p in microglia controls the development of schizophrenia-like symptoms in mice

Appendix:
Supplemental Figures 1 – 6

Appendix Fig. S1 + legend	Page 2
Appendix Fig. S2 + legend	Page 3
Appendix Fig. S3 + legend	Page 4
Appendix Fig. S4 + legend	Page 5
Appendix Fig. S5 + legend	Page 6
Appendix Fig. S6 + legend	Page 7

Appendix Fig. S1.

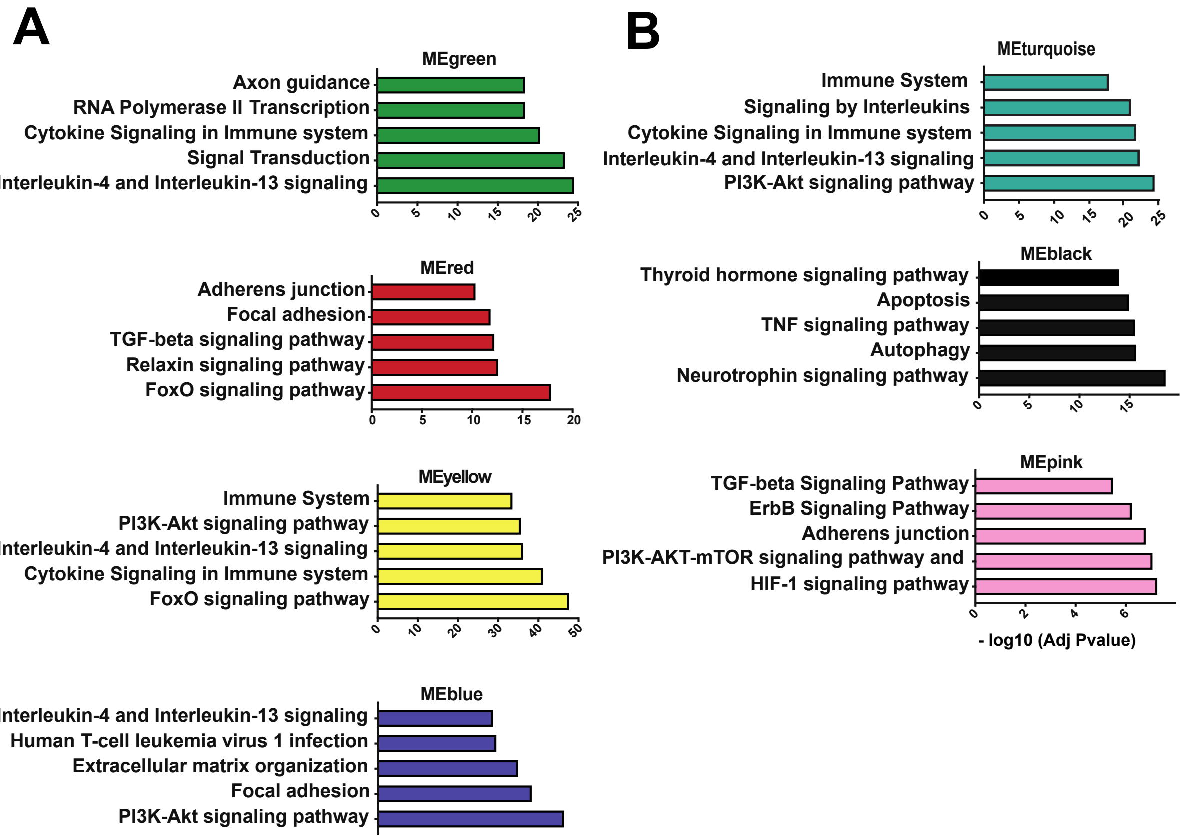

Appendix Fig. S1. GO-term analysis for confirmed mRNA targets of microRNAs found within the different co-expression modules. Confirmed mRNA targets were identified for the microRNAs within each of the detected co-expression modules (see Fig 1b). The corresponding gene-lists were subjected to GO-term analysis. **A.** Bar graphs depicting the top 5 GO-terms for the 4 co-expression modules (MEgreen, MEred, MEyellow and MEblue) that were increased in schizophrenia patients. **B. A.** Bar graphs depicting the top 5 GO-terms for the 3 co-expression modules (MEturquoise, MEblack, and MEpink) that were decreased in schizophrenia patients.

Appendix Fig. S2

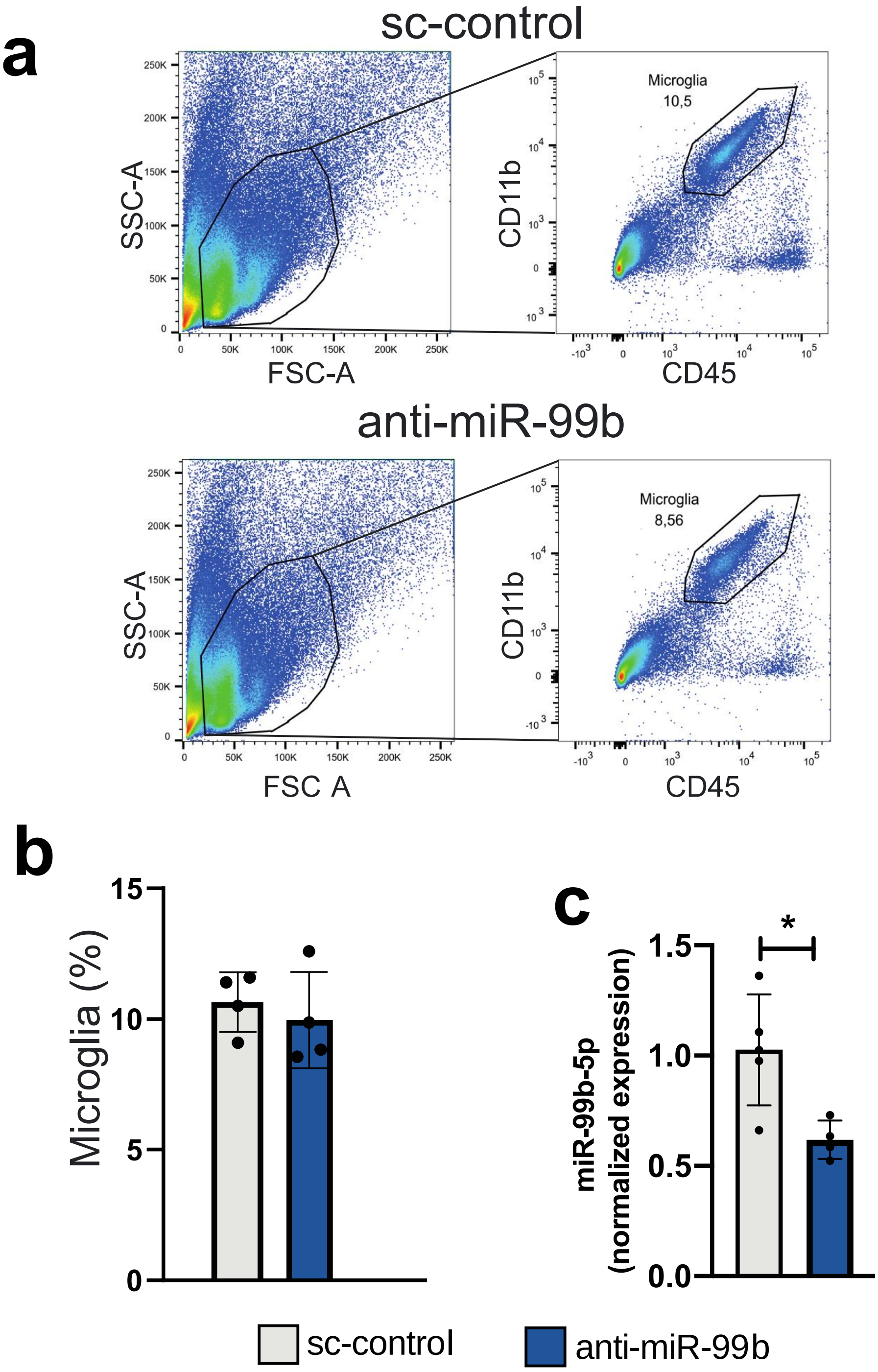

Appendix Fig. S2. Analysis of microglia obtained via FACS from the PFC of mice injected with anti-miR-99b or sc-control LNAs. **a.** Dot plots depicting the gating strategy for the isolation of $CD45^{low}/CD11b^{+}$ microglial cells from the PFC of sc-control or anti-miR-99b treated animals **b.** Corresponding quantification of microglia expressed as percentage of living cells. No statistical difference was observed between groups. **c.** Bar graph showing the expression of miR-99b-5p as determined via qPCR in PFC tissue isolated from mice injected into the PFC with sc-control LNAs or anti-miR-99b. Microglial miR-99b-5p levels are significantly reduced in mice that received anti-mIR-99b injection into the PFC. **P* < 0,05. Error bars indicate SEM.

Appendix Fig S3

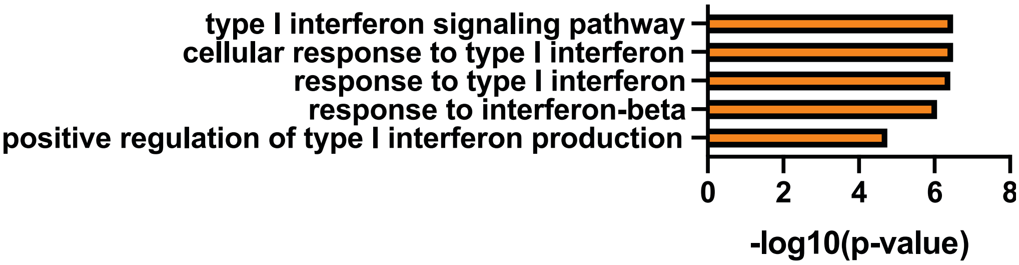

Appendix Fig. S3. GO-term analysis of miR-99b-5p target genes up-regulated in the PFC of mice treated with anti-miR99b. Bar graph depicting the 5 TOP GO-terms when analyzing miR-99b-5p target genes that were up-regulated in the PFC cortex of mice injected to the PFC with anti-miR-99b.

Appendix Fig. S4

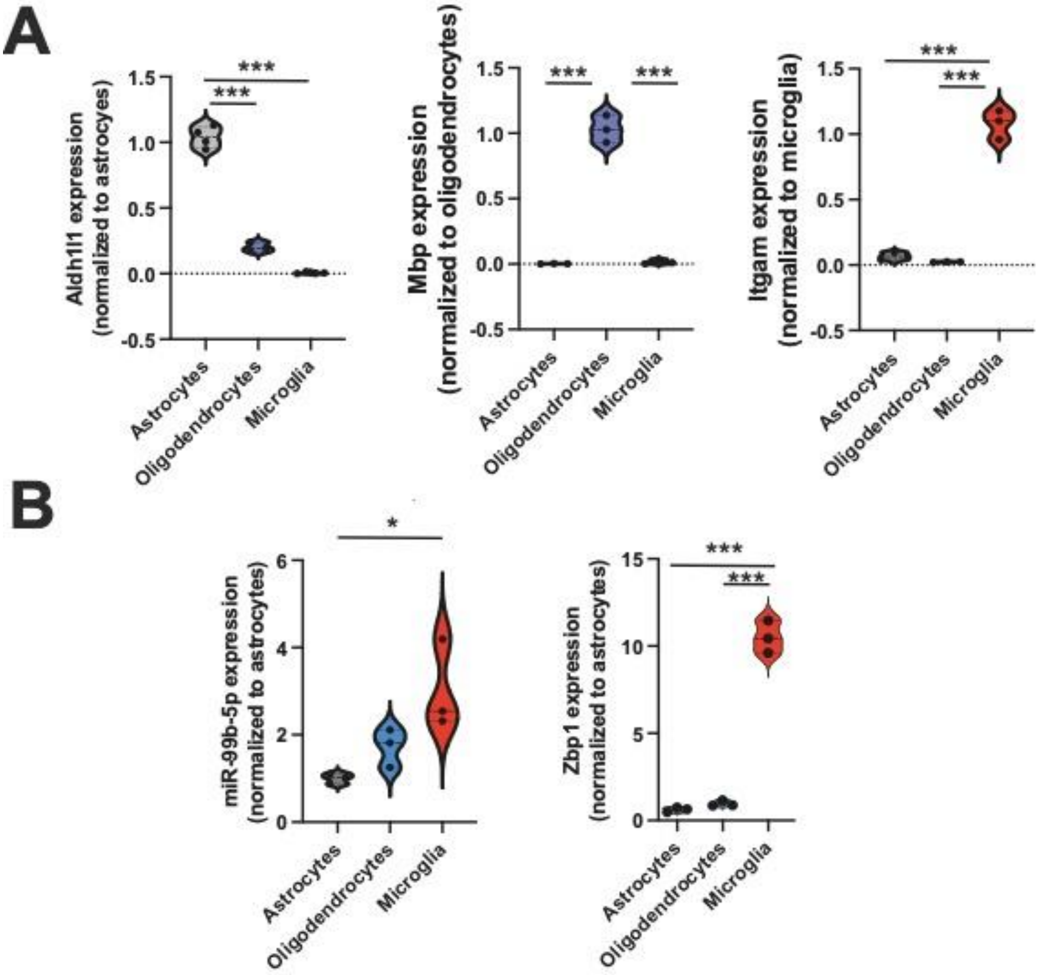

Appendix Fig. S4. Expression of miR-99b and Zbp1 in the mouse prefrontal cortex (PFC).
A. Magnetic-activated cell sorting (MACS) was used to isolate astrocytes, oligodendrocytes, and microglia from the PFC of 3-month-old male C57Bl6J mice. Cell type specificity was confirmed by qPCR using marker genes for astrocytes (*Aldehyde Dehydrogenase 1 Family Member L1, Aldh1l*), oligodendrocytes (*myelin basic protein, Mbp*), and microglia (*Integrin Subunit Alpha M, Itgam*). **B.** The expression of miR-99b-5p (left panel) and Zbp1 (right panel) was analyzed in MACS-isolated cells from the PFC of mice (same samples as in panel A). miR-99b-5p is enriched in microglia and significantly higher expressed than in astrocytes, while Zbp1 (right panel) is significantly higher expressed in microglia when compared to astrocytes and oligodendrocytes. Data was analyzed using One-way ANOVA followed by a t-Test. *$P < 0.05$, ***$P < 0.001$.

Appendix Fig. S5

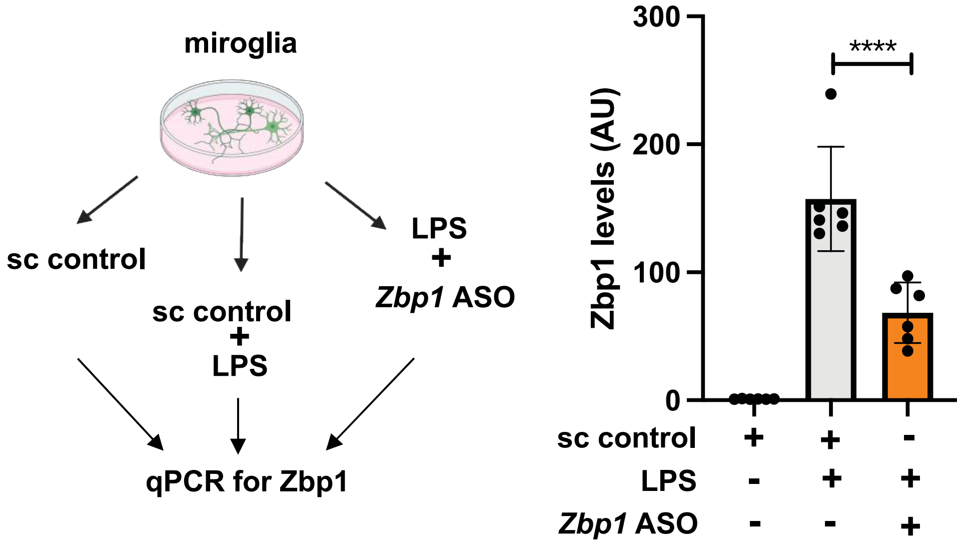

Appendix Fig. S5. *Zbp1* ASOs block LPS-induced Zbp1-expression in primary microglia. Left panel: Experimental design. Since *Zbp1* is expressed at low levels under physiological conditions, we decided to test the effect of the *Zbp1* ASOs in response to a strong immune stimulus, namely lipopolysaccharide (LPS) treatment. Primary microglia were treated with scrambled (sc)-control ASOs or ASOs (12.6 nM) targeting *Zbp1* (*Zbp1* ASO) 45h before treatment with LPS (100 ng/ml) for 3 hours. Cells were harvested and RNA was isolated for cDNA synthesis and qPCR. **Right panel:** Bar graph showing *ZBP1* mRNA levels measured via qPCR: We observed a strong increase of Zbp1 expression upon LPS treatment which was significantly reduced when cells were treated with Zbp1 ASOs (tTest, n= 6/group). ****P < 0.0001.

Appendix Fig. S6

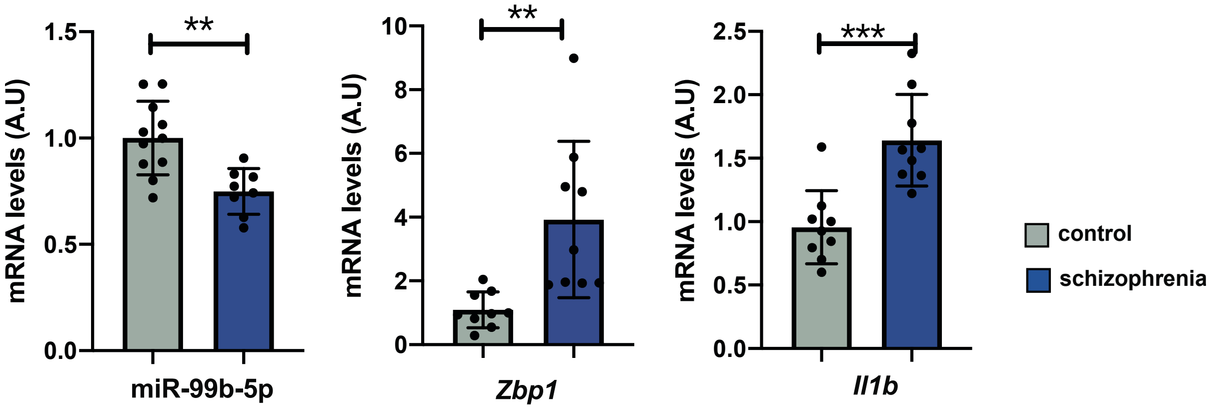

Appendix Fig. S6. Mir-99b-5p, Il1b and *Zbp1 expression in postmortem human brains*. Bar graph showing qPCR results for miR-99b-5p, *Zbp1* and *Il1b* in PFC tissue of SZ patients and control individuals. While miR-99b-5p levels are decreased, expression of the miR-99b-5p target gene *Zbp1* is increased in SZ patients. In line with this expression of the pro-inflammatory cytokine *Il1b*, which is regulated by *Zbp1*, is increased in SZ patients when compared to control. (*t*Test, ****P* < 0,001; ***P* < 0,01, **P* < 0,05; n = 9/group)
