# Supplementary figures and images for: Regulation of Zbp1 by miR-99b-5p in microglia controls the development of schizophrenia-like symptoms in mice
Source: EMBO J. 2024 Mar 25;43(8):3. doi: 10.1038/s44318-024-00067-8 (PMC11021462; doi:10.1038/s44318-024-00067-8)

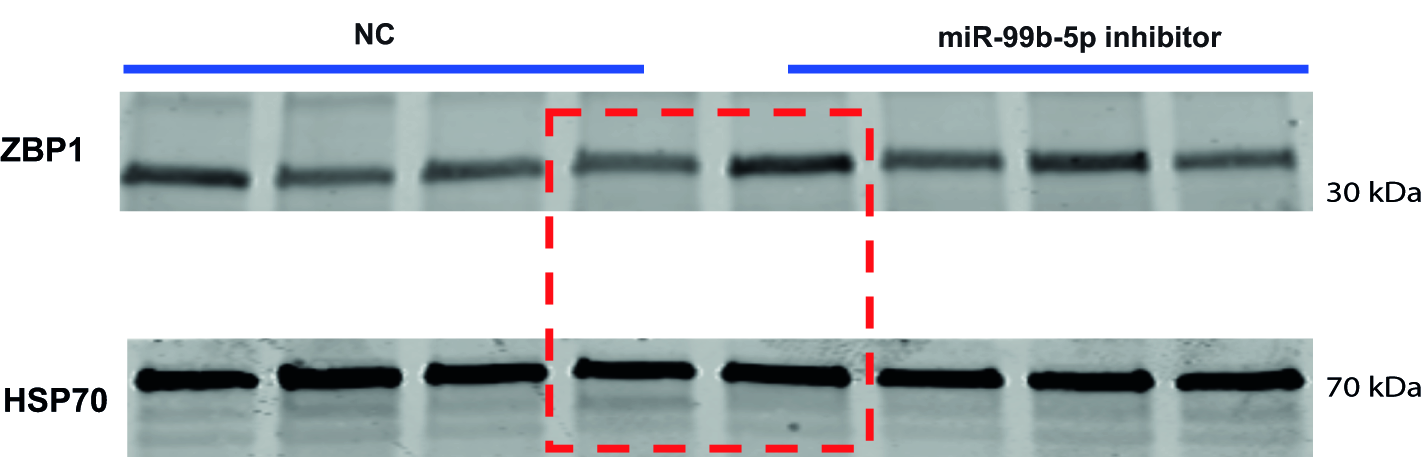

Supplement: Supplementary file 22 — Source data Fig. 4 [file 44318_2024_67_MOESM22_ESM.zip › Fig 4C/Immunoblot.tif]
