# Supplementary material for: Regulation of Zbp1 by miR-99b-5p in microglia controls the development of schizophrenia-like symptoms in mice
Source: EMBO J. 2024 Mar 25;43(8):3. doi: 10.1038/s44318-024-00067-8 (PMC11021462; doi:10.1038/s44318-024-00067-8)
Supplement: Supplementary file 25 — Expanded View Figures [file 44318_2024_67_MOESM25_ESM.pdf]

Expanded View Figures

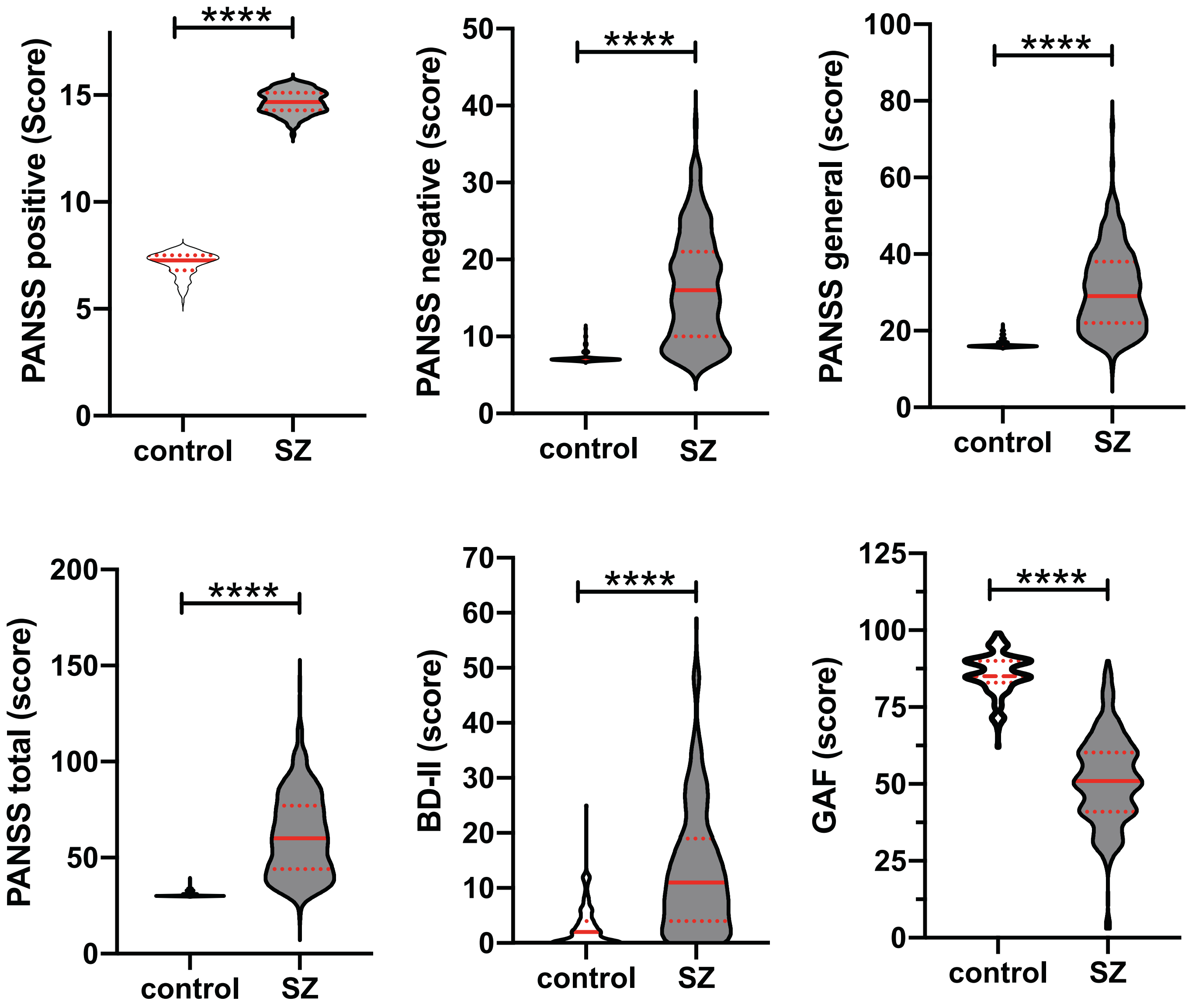

Figure EV1. Clinical phenotypes of the individuals subjected for small RNA-seq analysis.

We analyzed 242 healthy controls and 331 SZ patients of the PsyCourse study. Depicted are the clinical phenotypes that differ significantly between groups, namely the positive and negative syndrome rating scale (PANSS), the total PANSS, the Beck depression inventory (BDI-II) and the global assessment of functioning (GAF) scores. ****$P < 0.0001$, *t*Test.

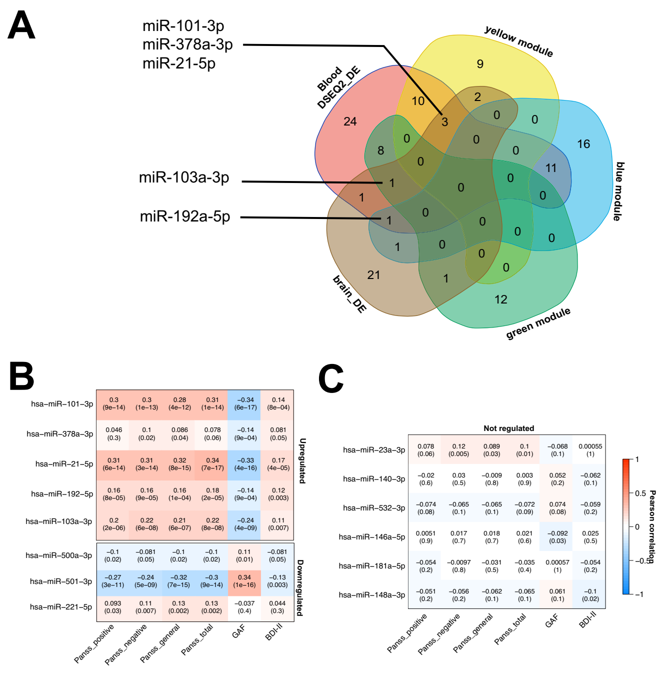

Figure EV2. Analysis of candidate miR expression to SZ phenotypes.

(**A**) Venn diagram showing the overlap between the miRs present within the co-expression modules that were increased in SZ patients (modules represented by yellow, blue and green color), that were increased when analyzed via DSEQ2 differential expression analysis in blood (Blood DSEQ2_DE) and were increased in postmortem brain samples from SZ patients (brain_DE). In total 5 miRs were consistently increased in blood, brain and a co-expression module (**B**) Heat map showing the correlation of candidate miR expression levels of individuals of the PsyCourse study to the clinical phenotypes. The numbers in each rectangle represent the correlation (upper number) and the corresponding p-value (lower number). Values for miR-99b-5p are shown within Fig. 1H. MiR-21-5p and miR-501-3p have been previously linked to SZ and show the highest correlation values. MiR-221-5p is significantly correlated to the PANSS scores but not the GAF and BDI-II (The significance of correlations was determined using Pearson correlation coefficients and assessed for statistical significance through permutation testing). (**C**) Heat map showing the correlation of six miRs from our dataset that were not differentially expressed in the blood or brain of SZ patients (not regulated). miR-23a-3p, miR-140-3p, and miR-532-3p were randomly selected, while miR-146a-5p, miR-181a-5p, and miR-148a-3p were recently identified as a biomarker signature for Alzheimer's disease (Islam et al, 2021) (The significance of correlations was determined using Pearson correlation coefficients and assessed for statistical significance through permutation testing).

 © The Author(s)

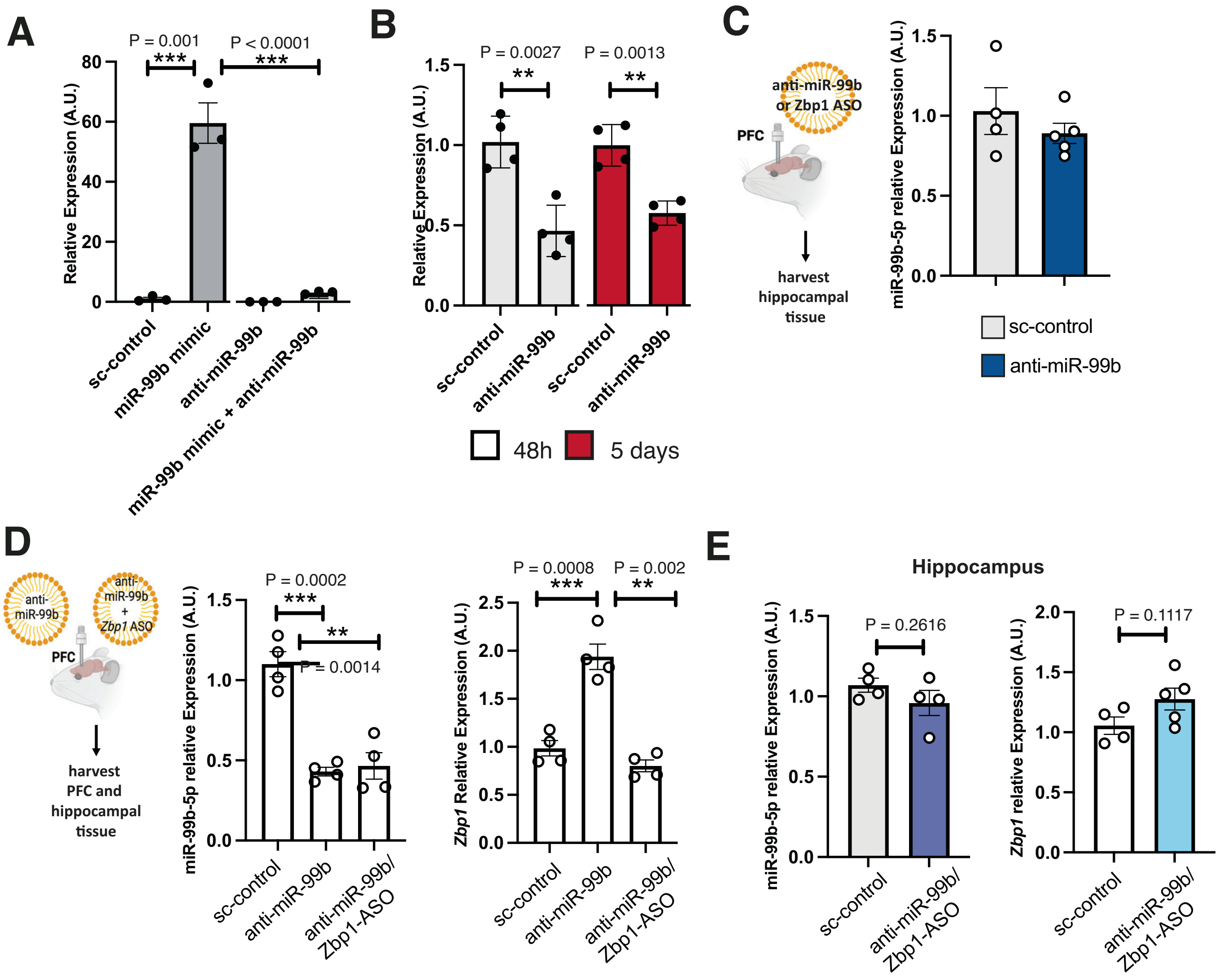

Figure EV3. Administration of anti-miR-99b-5p reduces the levels of miR-99b-5p detectable via qPCR.

(**A**) To test if the administration of anti-miR-99b oligonucleotides reduces the levels of miR-99b-5p detected via qPCR we treated HEK293 cells—which show low endogenous expression of miR-99b-5p—with either control oligonucleotides (sc-control), miR-99b mimic, anti-miR-99b or the combination of miR-99b mimic and anti-miR99b. While administration of the miR-99b mimic significantly increased the detection of miR-99b-5p via qPCR when compared to sc-control ($P = 0.001$, t-Test), the co-administration significantly reduced detectable miR-99b-5p ($P < 0.0001$, t-test). $N = 3$/group. Measurements were performed 48 after treatment. (**B**) Primary mouse microglia (DIV 8) were treated with sc-control or anti-miR-99b for either 48 h or 5 days before RNA was collected for qPCR. At both time points anti-miR-99b treatment significantly reduced the detectability of miR-99b-5p ($n = 4$/group, t-test). It is important to note that LNA-based anti-miRs are known to sequester the target RNA rather than causing the degradation of the target miR. Therefore, the reduced detection of miR-99b-5p upon anti-miR-99b treatment is likely due to the formation of exonuclease-resistant duplexes that are not denatured during cDNA synthesis. Consequently, the miR-99b-5p is less accessible for reverse transcription, resulting in lower detected levels compared to the scramble control-treated samples in the subsequent qPCR reaction. Nevertheless, qPCR is a suitable method to assay target engagement of anti-miR-99b-5p. (**C**) Left panel: Experimental design. Anti-miR-99b oligonucleotides (anit-mIR-99b) were injected—along with their corresponding control—into the prefrontal cortex (PCF) of mice. Right panel: Bar graph showing qPCR results for miR-99b-5p in tissue obtained from the hippocampus of mice after injection of anti-miR-99b ($n = 4$) or sc-control oligonucleotides ($n = 5$) to the PFC. There is no significant difference between the groups. (**D**) Left panel: Experimental design. Anti-miR-99b or anti-miR-99b along with *Zbp1*-ASOs (anti-miR99b/*Zbp1*-ASO) were injected into the prefrontal cortex (PCF) of mice. Middle panel: QPCR results from the PFC show that the expression of miR-99b-5p is decreased in the anti-miR-99b group ($n = 4$; $P = 0.0002$, unpaired t-Test) and in the anti-miR99b/Zbp1-ASO group ($n = 4$; $P = 0.0014$, unpaired t-Test) when compared to the sc-control group ($n = 4$). One-way ANOVA revealed a significant difference among the groups ($p < 0.0001$). Right panel: QPCR results from the PFC show that the expression of *Zbp1* is increased in the anti-miR-99b group ($n = 4$; $P = 0.0008$, unpaired t-Test) and in the anti-miR99b/Zbp1-ASO group ($n = 4$; $P = 0.002$, unpaired t-Test) when compared to the sc-control group ($n = 4$). One-way ANOVA revealed a significant difference among the groups ($p < 0.0001$). (**E**) Right panel: Bar graph showing qPCR results for *miR-99b-5p* in tissue obtained from the hippocampus of mice after injection of anti-miR-99b along with *Zbp1* ASOs (anti-miR99b/Zbp1-ASO group; $n = 4$) or control oligonucleotides ($n = 4$) into the PFC. There is no significant difference between the groups. Left panel: Bar graph showing qPCR results for *Zbp1* in tissue obtained from the hippocampus of mice after injection of anti-miR-99b along with *Zbp1* ASOs (anti-miR99b/Zbp1-ASO group; $n = 4$) or control oligonucleotides ($n = 5$) into the PFC. There is no significant difference between the groups. Data information: Bars and error bars in panels (**A–E**) indicate mean ± SEM.

© The Author(s)

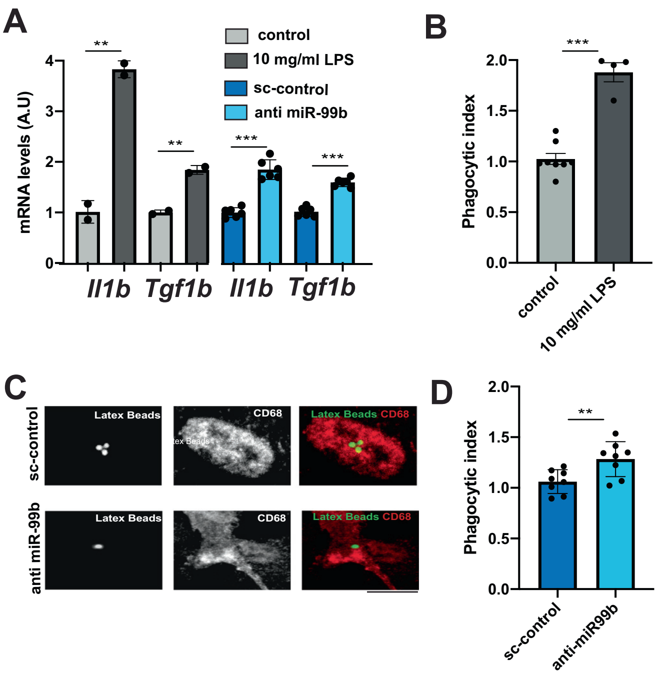

Figure EV4. Loss of miR-99b-5p levels increases the expression of pro-inflammatory cytokines and phagocytosis in IMG cells.

(**A**) IMG cells were treated with vehicle control solution (control) or 10 mg/ml LPS. In a similar experiment, IMG cells were treated with LNPs loaded with either sc-control LNAs or anti-miR-99b. qPCR analysis was performed for the pro-inflammatory cytokines *Il1b* and *Tgf1b*. The expression of *Il1b* and *Tgf1b* were significantly increased upon LPS treatment or anti-miR-99b treatment when compared to the respective control groups (unpaired t test; $n = 2$ or 6/group). (**B**) Bar graph showing that the phagocytic index increases in IMG cells upon LPS treatment (unpaired t test; controls = 8; LPS = 4). (**C**) Representative images showing the uptake of latex beads by IMG cells treated with either sc-control LNAs or anti-miR-99b. Scale bar: 0.5 µm. (**D**) Bar graph showing the quantification of (**C**). The figure presents representative images captured at 63× magnification using a confocal microscope. A scale bar of 0.5 µm is included in each image to provide a reference for size. Treatment with anti-miR-99 increases the phagocytic index (unpaired t test; $n = 8$/group). *$P < 0.05$, **$P < 0.01$, ***$P < 0.001$. Error bars indicate SEM. Data information: Bars and error bars in panels (**A**, **B**, **D**) indicate mean ± SEM.

 © The Author(s)

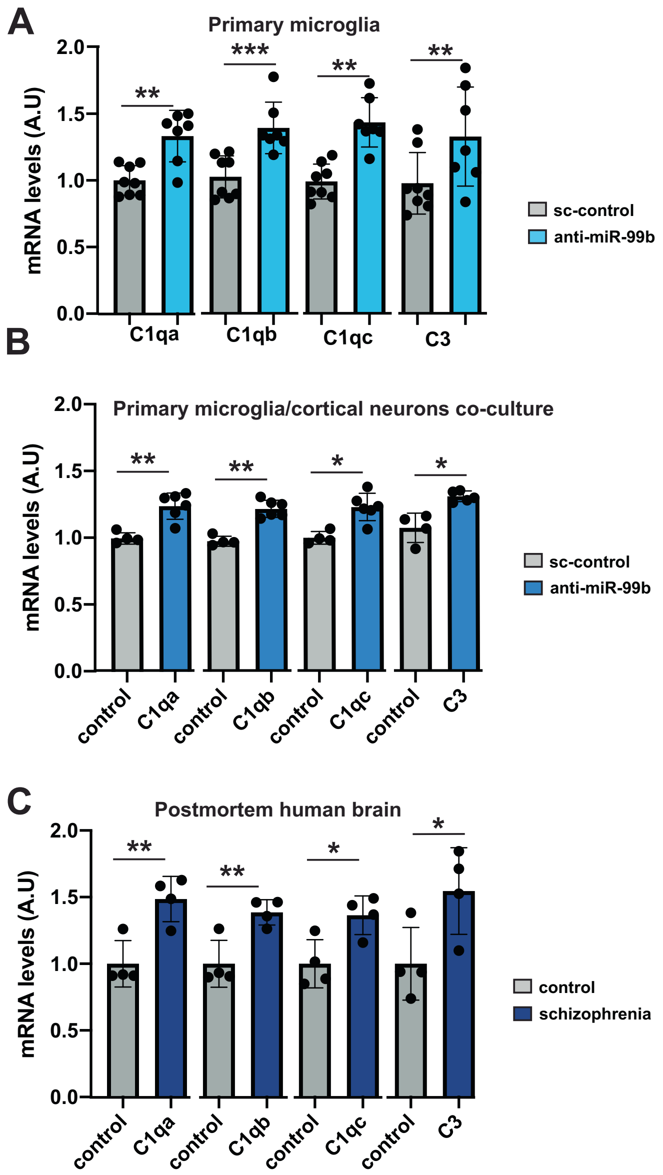

Figure EV5. Genes linked to innate immunity and synaptic pruning are increased in microglia treated with anti-miR-99b and in schizophrenia patients.

(**A**) Bar graph showing qPCR results for *C1qa*, *C1qb*, *C1qc*, and *C3* in primary microglia treated with sc-control LNAs or anti-miR-99b. (**B**) Bar graph showing qPCR results for *C1qa*, *C1qb*, *C1qc*, and *C3* when RNA was isolated from co-cultures in which primary cortical neurons were treated with microglia that had received LNPs loaded with either sc-control LNAs or anti-miR-99b. (**C**) QPCR analysis was used to measure *C1qa*, *C1qb*, *C1qc*, and *C3* expression in human postmortem PFC samples obtained from control individuals and SZ patients (unpaired *t*Test, $**P < 0.01$, $*P < 0.05$; $n = 4$/group). Data information: Bars and error bars in panels (**A**, **B**, **C**) indicate mean ± SEM.

© The Author(s)
